# Reliability and relative validity of a food frequency questionnaire to assess food group intakes in New Zealand adolescents

**DOI:** 10.1186/1475-2891-11-65

**Published:** 2012-09-05

**Authors:** Jyh Eiin Wong, Winsome R Parnell, Katherine E Black, Paula ML Skidmore

**Affiliations:** 1 Department of Human Nutrition, University of Otago, Dunedin, 9054, New Zealand; 2 School of Healthcare Sciences, Faculty of Health Sciences, Universiti Kebangsaan Malaysia, Kuala Lumpur, 50300, Malaysia

**Keywords:** Food frequency questionnaire (FFQ), Validity, Reproducibility, Reliability, Adolescents, New Zealand

## Abstract

**Background:**

Due to the absence of a current and validated food frequency questionnaire (FFQ) for use in New Zealand adolescents, there is a need to develop one as a cost-effective way to assess adolescents’ food patterns. This study aims to examine the test-retest reliability and relative validity of the New Zealand Adolescent FFQ (NZAFFQ) to assess food group intake in adolescents aged 14 to 18 years.

**Methods:**

A non-quantitative (without portion size), 72-item FFQ was developed and pretested. Fifty-two participants (aged 14.9 ± 0.8 years) completed the NZAFFQ twice within a two-week period for test-retest reliability. Forty-one participants (aged 15.1 ± 0.9 years) completed a four-day estimated food record (4DFR) in addition to the FFQs to enable assessment of validity. Spearman’s correlations and cross-classification analyses were used to examine relative validity while intra-class correlations were additionally used for test-retest reliability.

**Results:**

Weekly intakes were estimated for each food item and aggregated into 34 food groups. The median Spearman’s correlation coefficient (SCC) between FFQ administrations was 0.71. SCCs ranged from 0.46 for *fruit juice or cordial* to 0.87 for *non-standard milk*. The median intra-class correlation coefficient (ICC) between FFQ administrations was 0.69. The median SCC between food groups from the FFQ and the 4DFR was 0.40 with the highest SCC seen for *standard milk* (0.70). The exact agreement between the methods in ranking participants into thirds was highest for *meat alternatives* (78%), but lowest for *red or yellow vegetables* and *potatoes* (27%). The mean percent of participants misclassified into extreme thirds for food group intake was 12%.

**Conclusions:**

Despite a small sample size, the NZAFFQ exhibited good to excellent short-term test-retest reliability and reasonable validity in ranking the majority of the food group intakes among adolescents aged 14 to 18 years. The comparability of the validity to that in the current literature suggests that the NZAFFQ may be used among adolescent New Zealanders to identify dietary patterns and rank them according to food group intake.

## Background

The diet of adolescents, which is long known to be important for their growth and development, is now recognized as also important to their future health [1]. It is a major modifiable risk factor in the prevention of obesity and development of chronic diseases such as cardiovascular disease and cancer in adulthood [2,3]. The diet of adolescents in Western countries has been frequently described as being poor, with low consumption of dairy, fruits, vegetables and grains and high consumption of soft drinks and sweets [4-8]. Taken together, these issues heighten the need to accurately and reliably assess the food intake of adolescents, so as to allow for assessment of dietary patterns and diet quality in relation to future education and intervention. In order to do this appropriate methods for collecting dietary information from adolescents are needed. In New Zealand, information on the dietary intakes of adolescents has been collected in national surveys using in-depth measures such as a 24-hour dietary recall in combination with a food frequency questionnaire which had been tested for repeatability only [9]. However, the cost of such methods makes them prohibitive for use in all large scale studies. Therefore other suitable methods of obtaining dietary information from New Zealand adolescents are needed.

Considerations must be made when selecting appropriate dietary assessment methods for adolescents. Although most adolescents possess the literacy skills necessary for reliable self-reporting, accuracy of dietary assessment in this age group is affected by factors such as motivation to complete assessments and reporting bias associated with unstructured eating patterns, concerns with body image and weight status [10-12]. Besides these adolescent-specific issues, the study design, outcomes of interest and available resources need to be taken into consideration when selecting an appropriate dietary assessment tool for a study [13,14].

Food frequency questionnaires (FFQs), being relatively easy to administer and less onerous than other dietary assessment methods, appear to be a practical and affordable method for studying diets of adolescents [15]. They have been used successfully in large population studies and have been found to be valid and reliable tools for ranking food intakes of adolescents [16-18]. The major limitation of an FFQ lies in the measurement errors pertaining to an incomplete food list and inaccuracies in frequency and portion size estimation. In particular, the complex cognitive process of portion size estimation may pose additional challenges to adolescents who consume varying portion sizes across meals [19] and are less likely to pay attention to portion sizes than adults [10]. Although quantification skills may improve with intensive training and the use of age-appropriate food photograph aids [20,21], inclusion of portion size questions in an FFQ may increase respondent burden and lead to data omission, and hence contribute only marginally beyond frequency data in improving validity of an FFQ [22,23]. Therefore, recent research in this area has focused on the development of non-quantitative FFQs (without collection of portion size information) as targeted dietary assessment tools to rank individuals by intake of specific food groups, nutrients or dietary patterns rather than providing absolute values for foods and/or nutrients [13,24]. Besides providing information on usual intakes of a particular food or food groups of interest, such FFQs are particularly useful in identifying dietary patterns at the population level [25,26].

In addition to considering which type of FFQ (quantitative or non-quantitative) is most useful for a study, it is vital that any FFQ must be shown to be reliable and valid for use in the population of interest. An FFQ should also be designed to meet the aims of specific study populations and contain an up-to-date list of foods [27]. Although some FFQs exist for use in adolescents [18,28], they contain extensive food lists (more than 100 items) and portion size questions, which may not be relevant to the New Zealand context.

As there is currently no reliable, valid and up to date FFQ for use in New Zealand adolescents the aims of this study were to: (i) adapt an FFQ to assess food group intakes in New Zealand adolescents aged 14 to 18 years for use in future studies; (ii) determine short-term reliability of this FFQ, and (iii) determine the relative validity of this FFQ compared to an estimated food record.

## Methods

### Development of the New Zealand Adolescent FFQ (NZAFFQ)

This study was approved by the University of Otago Human Ethics Committee. A paper-based, three section adolescent-specific food questionnaire was developed. The food questionnaire is made up of three sections: The first section contains 12 multiple-choice questions on general eating habits, including intakes of food group servings, meal consumption patterns and frequency of takeaway consumption. These questions were adapted from previously published questionnaires [29,30]. This study focusses on the validity of the FFQ (sections 2 and 3 of the food questionnaire), namely the New Zealand Adolescent FFQ (NZAFFQ).

The NZAFFQ was produced by combining and modifying the Health Behaviour in School-aged Children (HBSC) FFQ [24] and the Children’s Dietary Questionnaire (CDQ) [26]. These FFQs were developed to describe food patterns, but not nutrient intakes, of children and adolescents (4 to 16 years), and therefore contain only a limited list of food items. In particular, the HBSC FFQ included 15 items covering the most commonly consumed foods known to be important sources of fibre and calcium among European youth. The CDQ included 28 items described as ‘encouraged foods’ (fruits, vegetables, water, reduced fat products) and ‘discouraged foods’ (high fat or sugar foods, sweetened beverages and full fat dairy products) for adolescents in Australia. These two validated questionnaires formed the basic construct of the NZAFFQ as they covered different important aspects (i.e. variety and intake frequency) of an adolescent’s diet and have been used to derive index-based dietary patterns [26,31,32].

Section 2 of the food questionnaire assessed ‘usual consumption’ of 32 food items, covering 15 items from the HBSC FFQ [24] and included extra questions on food groups relevant to the New Zealand adolescent population. Changes to the original HBSC FFQ included the addition of questions on consumption of meats and different types of soft drinks. As in the original HBSC FFQ, frequency of intake was estimated by asking “On average, how many times a week do you usually eat or drink……” and participants could select one of the following response categories: ‘none’, ‘less than once a week’, ‘once per week’, ‘2 to 4 days a week’, ‘5 to 6 days a week’, ‘once a day’, and ‘more than once a day’. Although a specific time frame for ‘usual’ was not defined, we believed that this was likely to cover the period of the previous four weeks, based on results of our pretesting group interviews. For the last section of the NZAFFQ (Section 3), we assessed intakes of 13 fruits, 22 vegetables and 7 miscellaneous foods consumed in the past seven days, as in the original CDQ [26]. Modifications to the original format included renaming and regrouping of conceptually similar food items to ensure the suitability of the food items to New Zealand. For example, ‘sweet potato’ was renamed as the locally known Māori name ‘kumara’ while ‘orange’ and ‘mandarin’ were grouped together as one item. Section 3 assessed ‘most current intake’ (in the past week) to reduce the difficulty of recall and accommodate seasonality and availability of foods [33].

Further revisions were also made to ensure that the food lists in sections 2 and 3 covered the foods frequently consumed in New Zealand including those indicated in the 2002 National Children’s Nutrition Survey [9]. To improve face validity of the NZAFFQ, two registered dietitians and a nutritionist were consulted to review this FFQ before formal pretesting.

### Pretesting of the NZAFFQ in group interviews

The NZAFFQ was pre-tested in a sample of 29 adolescents (13 males, 16 females) aged 14 to 15 years recruited from one secondary school in Dunedin, Otago. The day before the pretesting, participants were asked to complete the FFQs and answer feedback questionnaires about their comprehension of the NZAFFQ questions. Two group interviews (one for males, one for females) were conducted, each moderated by a trained research assistant, with additional observers present to identify further discussion questions based on feedback from the group. The main focus of the group interview was to obtain details on the participants’ understanding of the questions, the food items listed and examples. The group discussions were audio recorded and transcribed so that the feedback could be used to further refine the FFQ. The final NZAFFQ included 74 food items as either (i) single foods (e.g. yoghurt), or (ii) lists of similar foods (e.g. chicken, turkey or duck). For analysis, these foods were subsequently aggregated into 34 food groups of interest by grouping similar items as had been done in other studies [34,35] (Table 1).

**Table 1:** Food grouping for data analyses

	***Food groups***	**N**^**a**^	**Food items in the NZAFFQ**
1	*Fruit juice or cordial*	1	Fruit juice / drink / cordials
2	*Artificially sweetened drink*	1	Artificially sweetened drinks
3	*Tea or coffee*	1	Tea or coffee (including Ice Tea)
4	*Milky or chocolate drink*	1	Milky or chocolate drink
5	*Sugar-added drinks*	3	Regular soft drinks, Sports drink, Energy drinks
6	*Breakfast cereals*	1	Breakfast cereals (all kinds)
7	*Non-white bread or bun*	1	Brown or wholegrain bread or roll
8	*Rice, pasta or noodles*	1	Rice / pasta / noodles
9	*White bread or bun*	1	White bread or roll
10	*Cheese*	1	Cheese
11	*Non-standard milk*	1	Low-fat milk (light blue)/ Trim milk (green)/ Calci Trim milk (yellow)/ Rice milk/ Soy milk
12	*Standard milk*	1	Standard milk (dark blue)
13	*Yoghurt*	1	Yoghurt
14	*Poultry*	1	Chicken/ turkey/duck
15	*Eggs*	1	Eggs
16	*Nuts or seeds*	1	Nuts or seeds
17	*Meat alternatives*	1	Tofu /vegetarian sausages /falafel
18	*Legumes*	1	Baked beans/ chickpeas/ lentils/ kidney beans
19	*Red meat and processed meat*	4	Beef, Lamb or mutton, Pork, Processed meat (including sausage, salami and luncheon)
20	*Fish and seafood*	2	Fish, Other seafood (including mussels, oyster, prawns)
21	*Fruits*	13	Apple, Banana, Oranges or mandarins, Peaches or nectarines, Pears, Apricots, Plums, Kiwifruit, Strawberries or berries, Grapes, Melons (including watermelon, rockmelon, honeydew), Pineapple, Avocado
22	*Cruciferous vegetables*	3	Broccoli or cauliflower, Cabbage or coleslaw, Brussels sprouts
23	*Green leafy vegetables*	4	Lettuce or salad green, Mixed vegetables, Watercress or puha, Silverbeet or spinach
24	*Marrow-like vegetables*	2	Cucumber, Zucchini or courgette
25	*Red or yellow vegetables*	5	Pumpkin, Kumara, Carrots, Capsicums, Tomatoes
26	*Potatoes*	2	Hot chips or wedges or French fries, Potatoes (not fried)
27	*Other vegetables*	6	Onion or leeks, Mushrooms, Corn, Taro, Peas or green beans, Celery or asparagus.
28	*Sweet bakery products*	1	Sweet biscuits/ cakes/ muffins/ doughnuts/ fruit pies
29	*Sweet snack bars*	1	Muesli bar / fruit bar / rice bubble bar
30	*Nut spread*	1	Peanut butter / nut spread
31	*Ice-cream*	1	Ice-cream
32	*Sweets*	2	Lollies, Chocolate confectionery
33	*Convenience foods*	2	Pies or sausage rolls, Pizza
34	*Savoury biscuits and crisps*	2	Potato crisps or corn snacks, Savoury biscuits or snacks

Abbreviation: New Zealand Adolescent Food Frequency Questionnaire (NZAFFQ).

^a^ Number of items in the NZAFFQ. Item 'Alcoholic drink' was not included in the analysis.

### Validation of the NZAFFQ

A convenience sample of adolescents aged 14 to 18 years was recruited to participate in the validation study via schools, sports clubs and youth groups in Dunedin based on the following inclusion criteria: aged between 14 and 18 years, absence of any disease that may influence nutritional status, ability to keep a food record, and those whose parents did not complete an opt-out consent form. All participants also provided written informed consent before participating in the study. Data collection was conducted in two periods from November to early December 2010 and March to May 2011.

### NZAFFQ and estimated food record

Each participant was asked to keep a four-day estimated food record (4DFR) and complete the NZAFFQ twice within a fortnight. On the first visit day, each participant was asked to self-complete an FFQ in the presence of a research assistant. After the FFQs were checked for completeness and missing answers were obtained, participants were given verbal and written instructions on how to complete a structured Food and Drink diary for three weekdays and one weekend day. To facilitate accurate recording, the instructions incorporated standardized examples on the methods of recording and the food record was structured into six daily eating occasions. On each page, spaces were provided for recording of meal times, venue, whom the participants ate with, details of foods consumed including type, brand and amount of foods or beverages consumed. In addition, each participant was taught to use the portion aid kit provided for recording food quantities. The portion aid kit included a metric measuring cup, ruler, diameter circle and a coloured food photo booklet, which contained photographs of commonly consumed foods in different portion sizes [36]. At the end of the four-day record, participants answered supplementary questions on the type of sweetened drink, milk, bread and fat spread that they usually consumed. This qualitative information was collected to assist with coding of the food records. All food records received were checked for completeness. Participants were asked to clarify missing and unclear entries whenever possible. Within two weeks after the first completion of the NZAFFQ, all participants were asked to repeat the NZAFFQ for a second time to assess test-retest reliability. Based on results of previous research of FFQ repeatability conducted in New Zealand children [37], this two-week interval was chosen in order to minimize the variation in food intake responses due to true changes over time.

### Data and statistical analysis

All data were entered into a Microsoft Excel spreadsheet and checked by a trained nutritionist. Recorded food items in the 4DFR were matched to the items as defined by the NZAFFQ. An example of this was ‘hash browns’ from the 4DFR assigned to ‘fried potatoes’ in the NZAFFQ. Food items within a composite meal were each allocated a proportion of the meal then assigned to their relevant food or food groups. For instance, a chicken burger was recorded to the three corresponding foods in the NZAFFQ: white bread/bun, poultry and lettuce/salad greens. All frequencies obtained from the 4DFR were adjusted to provide average weekly intakes. Food intakes were described as frequencies of intake (days per week) and used as the unit of comparison between the NZAFFQ and the 4DFR.

Spearman’s rank correlations were performed to evaluate the validity of the first FFQ administration (FFQtime1) relative to the 4DFR for ranking participants by frequency of consumption of the 34 food groups. Following grouping of participants into thirds, cross-classification analyses were undertaken to examine the proportion of participants correctly classified into the same thirds or grossly misclassified into extreme thirds of intake. For reliability (FFQtime1 vs. FFQtime2), intraclass correlation coefficients (ICCs) were calculated in addition to Spearman’s correlation analyses. Because ICCs take account of within- and between-subject variability in responses, it was deemed the most appropriate test to examine the agreement between the repeated FFQs in ranking individuals by food group intakes. All statistical analyses were performed using the statistical program STATA 11.1 (StataCorp, College Station, TX, USA). Significance levels for all tests were set at p < 0.05.

## Results

### Sample

Of the 78 participants who volunteered to take part in this study, 38 (49%) participants completed both the 4DFR and two replications of the NZAFFQ. Fourteen participants (18%) completed two NZAFFQs only while three participants (4%) completed the 4DFR and the FFQtime1. More males than females did not complete all parts of this study (p = 0.006) but there were no differences in demographic or anthropometric data between those who completed all parts of the study and those who did not (data not shown). In total, 41 participants (16 males, 25 females) were included in the validity study while 52 participants (28 males, 24 females) were included in the reliability study. Participants were aged 15.0 ± 0.8 years (range 14.0 to 17.9 years). The mean time interval between test-retest administrations of the NZAFFQ was 12 days.

### Test-retest reliability (FFQtime1 vs. FFQtime2)

The median Spearman’s correlation coefficient (SCC) between the two administrations of the NZAFFQ was 0.71, and SCCs ranged from 0.46 for *fruit juice or cordial* to 0.87 for *non-standard milk* (Table 2). The median ICC was 0.69 (range 0.26-0.92) and 71% (24 of 34) of the food groups had ICCs above 0.6. At least 46% of participants were correctly classified into the same thirds for all food groups. No food groups had levels of gross misclassification above 10 percent, with the exception of *meat alternatives* (17%) and *rice, pasta or noodles* (25%).

**Table 2:** Test-retest reliability and relative validity of the New Zealand Adolescent Food Frequency Questionnaire (NZAFFQ): Spearman’s correlation coefficients, intraclass correlation coefficients, percent correctly classified and grossly misclassified into thirds of food group intake

	**Test-retest reliability (n** = **52)(FFQtime1 vs. FFQtime2)**				**Relative validity (n = 41) (FFQtime1 vs. 4DFR)**		
**Food Groups**	**SCC**	**ICC**	**% CC**	**% GM**	**SCC**	**% CC**	**% GM**
*Fruit juice or cordials*	0.46	0.57	61	10	0.40	46	10
*Artificially sweetened drinks*	0.82	0.68	80	4	0.25	56	24
*Tea or coffee*	0.85	0.70	88	4	0.43	63	20
*Milky or chocolate drinks*	0.75	0.81	67	2	0.62	46	5
*Sugar-added drinks*	0.77	0.37	71	4	0.32	49	17
*Rice, pasta or noodles*	0.47	0.80	75	25	0.44	37	17
*Non-white bread or bun*^1^	0.80	0.72	61	4	0.36	45	13
*Breakfast cereals*	0.78	0.74	56	0	0.67	56	10
*White bread or bun*	0.78	0.64	73	4	0.40	37	10
*Cheese*	0.67	0.58	58	2	0.40	41	10
*Non-standard milk*^2^	0.87	0.79	78	0	0.59	55	8
*Standard milk*^3^	0.83	0.78	67	0	0.70	61	2
*Yoghurt*	0.79	0.84	75	0	0.46	54	10
*Poultry*	0.63	0.34	69	6	0.46	51	7
*Eggs*	0.80	0.53	77	0	0.52	41	5
*Nuts or seeds*	0.71	0.73	71	6	0.24	54	22
*Meat alternatives*	0.54	0.92	83	17	0.55	78	22
*Legumes*	0.50	0.45	53	6	0.42	59	17
*Red meat and processed meat*	0.60	0.26	62	8	0.13	41	15
*Fish and seafood*	0.76	0.67	65	2	0.34	41	15
*Fruits*	0.58	0.83	58	8	0.27	44	10
*Cruciferous vegetables*	0.72	0.64	63	4	0.26	46	20
*Green leafy vegetables*	0.74	0.77	69	4	0.27	44	17
*Red or yellow vegetables*	0.60	0.70	46	2	0.08	27	12
*Marrow-like vegetables*	0.59	0.76	56	6	0.39	59	17
*Potatoes*	0.56	0.61	71	6	0.25	27	7
*Other vegetables*	0.65	0.56	60	4	0.40	59	10
*Sweet bakery products*^4^	0.65	0.62	67	10	0.56	44	12
*Sweet snack bars*^5^	0.65	0.56	60	4	0.58	61	5
*Nut spreads*	0.79	0.73	73	2	0.37	59	20
*Ice-cream*	0.64	0.79	58	4	0.42	51	5
*Sweets*^6^	0.79	0.54	77	2	0.18	39	20
*Convenience foods*^7^	0.56	0.67	67	10	0.04	46	29
*Savoury biscuits and crisps*	0.70	0.70	62	4	0.29	39	12

Abbreviation: first administration of the New Zealand Adolescent Food Frequency Questionnaire (FFQtime1), second administration of the New Zealand Adolescent Food Frequency Questionnaire (FFQtime2), four-day estimated food records (4DFR), Spearman’s correlation coefficients (SCC), intraclass correlation coefficients (ICC), percent correctly classified (%CC), percent grossly misclassified (%GM).

^1^ Brown or wholegrain bread.

^2^ Low-fat milk, trim milk, calcium-fortified trim milk, rice milk, soy milk.

^3^ Whole-fat milk.

^4^ Sweet biscuits, cakes, muffins, doughnuts, fruit pies.

^5^ Muesli bars, fruit bars rice bubble bars.

^6^ Lollies, chocolate confectionery.

^7^ Pies, sausage rolls, pizza.

### Relative validity (FFQtime1 vs. 4DFR)

As shown in Table 2, SCCs above 0.3 were seen for over two-thirds (23) of the 34 food groups in the FFQ. The median SCC was 0.40, and individual SCCs ranged from 0.04 for *convenience foods* to 0.70 for *standard milk* (whole-fat milk)*.* High correlations (SCC ≥ 0.50) were observed for *breakfast cereals*, *milk (standard and non-standard)*, *eggs*, *sweet bakery products* and *sweet snack bars*. Overall, the exact agreement between the methods in ranking participants into thirds was highest for *meat alternatives* (78%), but lowest for *red or yellow vegetables* and *potatoes* (27%). The mean percent misclassified into extreme thirds for all food groups was 12%.

## Discussion

In the present study, the short-term reliability of a non-quantitative FFQ (NZAFFQ) was established by comparing two administrations of the FFQ over a two-week period while relative validity was established against a 4DFR.

The results of this study demonstrated that the NZAFFQ yielded good test-retest reliability. The median ICC of 0.69 (range 0.26-0.92) compared favorably to those reported in previous studies in adolescent populations (ICC range 0.01-0.83) [38-40]. The median SCC was 0.71, with all food groups achieving Spearman’s correlations above 0.46. This reliability fell within a range considered good for an FFQ (0.50–0.80) [27,41] and was similar to the reliability of the CNS02 FFQ, the only previous FFQ designed for New Zealand children [37]. The median test-retest correlation for the CNS02 FFQ for food servings was 0.73, ranging from 0.54 for mixed meat dishes to 0.89 for convenience meals in the 10–14 year age group (n = 42). As indicated by the ICC, we found that foods that were consumed regularly (e.g*. milky or chocolate drinks*) were recalled with more consistency than foods that were consumed occasionally or variably (e.g. *red meat, processed meats* and *poultry*), as observed in previous studies [17,42,43]. We acknowledge that the two-week interval between the administration of the NZAFFQs may have led to overestimation of the reliability of this FFQ.

Some variation was seen in the levels of validity between food groups. Among 34 food groups, most food groups (67%) yielded SCCs between 0.32 and 0.70 while 11 food groups produced correlations below 0.30. In particular, the NZAFFQ was less accurate in estimating the group intakes of some vegetables (*cruciferous, green leafy, red or yellow vegetables and potatoes*), *fruits* and *red meat and processed meat*. There are several possible explanations for this observed poor validity for these food groups.

Firstly, within-participant intakes of fruits, vegetables and meats were shown to be highly variable [44]. It is therefore possible that some of the food items consumed occasionally or episodically were not being consumed during the four-day recording period. This is a known limitation when a reference method that covers only a limited period of time is used to validate an FFQ [45]. Notably, 64% of vegetables, 85% of fruits and 64% of meat groups were consumed ‘once a week or less’ by more than two-thirds of the participants. These foods each had a 54% chance of not being consumed during the recording period. Although extending the number of recording days may potentially improve the correlations, this would have caused reporting fatigue and reduced the quality and completion of the food records [19].

Secondly, we noted that different recording methods and time frame might have attenuated the correlations between the NZAFFQ and the 4DFR. For the NZAFFQ, a particular food eaten both alone and in mixed dishes was recorded in a combined frequency. Conversely for the 4DFR, mixed dishes were recorded then segregated into their component foods and apportioned to their matching food groups. Because of this, foods often consumed as part of mixed dishes such as *red or yellow vegetables* (e.g. tomatoes and capsicum), *red meat* and *processed meat* (e.g. sausage) may either be forgotten (thus underestimated in the NZAFFQ) or miscoded in the food records due to insufficient information [7,46]. In addition, following recommendations by Cade and colleagues [27], the FFQtime1 was administered before the 4DFR to eliminate learning effects from completion of a more onerous dietary method. As the NZAFFQ asked about ‘past seven days’ intakes for fruits and vegetables (in Section 3), it assessed diet retrospectively over a slightly different time span from the reference method. These issues of methodological difference between the two dietary methods (i.e. coding decisions and reference period) may have had a negative impact on the correlations of the food group intakes. It is also important to acknowledge that using FFQtime1 in validity analyses could potentially result in an underestimation of validity.

### Comparison with other studies

Although validation studies of FFQs in adolescents have been previously reported, most studies validated their FFQs in terms of nutrients or absolute food intakes [47,48]. Since this study focused on validating the actual responses of intake frequency, precise comparisons of this study with existing studies are not possible. One exception is a study by Vereecken and Maes [24], which validated a 15-item HBSC FFQ against a 7-day food diary using a similar approach to this study. These same 15 food items were included in the present NZAFFQ. A direct comparison of the two studies revealed that the NZAFFQ showed similar reliability (mean SCC = 0.71) compared to the original HBSC FFQ validation study (mean SCC = 0.67) for the 15 food items. Likewise, the validity of most food items was comparable to those of the original HBSC FFQ (mean SCC = 0.41), achieving an average SCC of 0.38. Vereecken and Maes found high correlations for milk (whole and semi-skimmed) and brown bread (SCC = 0.51-0.65) but low correlations for crisps, diet soft drinks, sweets and fruits (SCC = 0.10–0.34). Similarly, our study showed that validity was good for milk and breakfast cereals (SCC = 0.59-0.70), but less favorable for soft drinks (regular and diet), chips, crisps and sweets (SCC = 0.06-0.30). The most striking observation that emerged from the data comparison was the rather low validity for foods perceived as being ‘less healthy’. We speculate that these foods may be underreported in the NZAFFQ due to social undesirability [49]. This is evident for regular soft drinks where 50% of participants who reported usual consumption of ‘once or less per week’ in the NZAFFQ specified intake on two or more days during their 4-day recording periods.

### Strengths and limitations

This study has several strengths and limitations. The main limitation of this study was the small sample size (n = 41), which may have limited the observation of significant correlations in food group intakes. Previous authors have suggested that a sample size of at least 50 is desirable [27], and ideally a sample of between 100 and 200 should be used, particularly if the FFQ is designed to provide information on nutrient intakes [41]. Although the recruitment deadlines were shifted several times, it was difficult to recruit more participants. A high percent (47%) of those recruited failed to complete the study due to the demanding task of keeping a 4DFR, even though estimated rather than weighed records were used. Our low compliance rate fell within the response range of 48% to 60% typically observed in previous validation studies of adolescents [19,50,51]. The sample in our study may comprise participants who were highly motivated; hence generalizability of these findings to other adolescent populations in New Zealand may be limited. On the other hand, this reinforces the clear need to develop a simple FFQ to accurately assess diet among adolescents, including those who are unlikely to provide high quality food records.

In the absence of an absolute gold standard for dietary assessment, we chose an estimated food record as the reference method. This method is advantageous in its ability to capture all food intakes without the reliance of memory and hence has the fewest correlated errors with an FFQ [52]. Additional effort was taken to prepare the Food and Drink Diary as an easy-to-carry booklet to facilitate recording ‘in situ’. Although participants were instructed to conduct recording ‘at the time’ of food and beverage consumption, we acknowledge that this may not be entirely possible. Food underestimation may still occur due to forgetfulness and the limited food knowledge among adolescents [47,53,54]. In addition, as the present NZAFFQ also assesses food intakes in the past, the different time frame between the FFQs and the food records may have had an effect on the correlations. Nevertheless, we found similar correlations between this study and other studies with overlapped time frame [38,39,44].

The strength of this study lies in the design of a non-quantitative FFQ, which is relatively short and practical for use in time-limited surveys where detailed measures of food intakes are not feasible. We attempted to address the limited motivation and portion size estimation skills among adolescents by omitting the requirement to provide food quantities in the NZAFFQ. As a result, this FFQ was highly repeatable and could be self-completed within 15 minutes. The median SCC of 0.40 obtained from this study was comparable to other validation studies of quantitative FFQs in adolescent populations [38,44,50]. Encompassing a wide range of food items from different food groups, this FFQ may offer a viable approach to measure diet diversity and derive dietary patterns or diet quality indices in large studies of adolescents. Whilst the intended use of the NZAFFQ is to assess food group intakes of adolescents in New Zealand, there is a potential for the frequency data to be used alongside other more intensive dietary assessment methods such as the 24-hour diet recall to estimate usual intake [55]. As it was adapted from previously validated questionnaires and pretested rigorously, we believe that the food list sufficiently covers the common foods consumed by New Zealand adolescents and is hence suitable to assess food group intakes in this age group.

## Conclusions

Despite a small sample size, the NZAFFQ exhibited good to excellent test-retest reliability and reasonable validity in ranking intakes for a majority of the food groups. This positive finding raises the possibility that the true ability of the NZAFFQ to rank food intakes in adolescents is greater than that shown by our data. Based on the present study, we recommend that the NZAFFQ is appropriate for ranking participants according to food group intake and may be applied in future studies to assess dietary patterns of adolescents aged 14 to 18 years.

## Consent

All participants provided written informed consent before participating in the study. Parents were only required to provide opt-out consent on behalf of their child.

## Abbreviations

FFQ: Food frequency questionnaire; NZAFFQ: New Zealand Adolescent Food Frequency Questionnaire; FFQTime1: First administration of the NZAFFQ; 4DFR: Four-day estimated food records; HBSC: Health Behaviour in School-aged Children; CDQ: Children’s Dietary Questionnaire; SCC: Spearman’s correlation coefficient; ICC: Intra-class correlation coefficient; %CC: Percent correctly classified; %GM: Percent grossly misclassified.

## Competing interests

The authors declare that they have no competing interests.

## Authors' contributions

All authors were responsible for conception of the study, study design and set up. JW, KB and PS were responsible for data collection. JW entered and analyzed the data and drafted the manuscript. All authors were involved with data interpretation, critical revisions of the paper and provided approval for its publication. All authors read and approved the final manuscript.
